# Adipose tissue specific CCL18 associates with cardiometabolic diseases in non-obese individuals implicating CD4^+^ T cells

**DOI:** 10.1186/s12933-023-01803-w

**Published:** 2023-04-12

**Authors:** Narmadha Subramanian, Kaisa Hofwimmer, Beatriz Tavira, Lucas Massier, Daniel P Andersson, Peter Arner, Jurga Laurencikiene

**Affiliations:** Lipid laboratory, Unit of Endocrinology, Dept. of Medicine Huddinge, Karolinska Institutet, Stockholm, 141 86 Sweden

**Keywords:** Non-obese, T2D, CVD, CCL18, CD4^+^ T cells, Subcutaneous WAT

## Abstract

**Aim:**

Obesity is linked to cardiometabolic diseases, however non-obese individuals are also at risk for type 2 diabetes (T2D) and cardiovascular disease (CVD). White adipose tissue (WAT) is known to play a role in both T2D and CVD, but the contribution of WAT inflammatory status especially in non-obese patients with cardiometabolic diseases is less understood. Therefore, we aimed to find associations between WAT inflammatory status and cardiometabolic diseases in non-obese individuals.

**Methods:**

In a population-based cohort containing non-obese healthy (n = 17), T2D (n = 16), CVD (n = 18), T2D + CVD (n = 19) individuals, seventeen different cytokines were measured in WAT and in circulation. In addition, 13-color flow cytometry profiling was employed to phenotype the immune cells. Human T cell line (Jurkat T cells) was stimulated by rCCL18, and conditioned media (CM) was added to the *in vitro* cultures of human adipocytes. Lipolysis was measured by glycerol release. Blocking antibodies against IFN-γ and TGF-β were used *in vitro* to prove a role for these cytokines in CCL18-T-cell-adipocyte lipolysis regulation axis.

**Results:**

In CVD, T2D and CVD + T2D groups, CCL18 and CD4^+^ T cells were upregulated significantly compared to healthy controls. WAT CCL18 secretion correlated with the amounts of WAT CD4^+^ T cells, which also highly expressed CCL18 receptors suggesting that WAT CD4^+^ T cells are responders to this chemokine. While direct addition of rCCL18 to mature adipocytes did not alter the adipocyte lipolysis, CM from CCL18-treated T cells increased glycerol release in *in vitro* cultures of adipocytes. IFN-γ and TGF-β secretion was significantly induced in CM obtained from T cells treated with CCL18. Blocking these cytokines in CM, prevented CM-induced upregulation of adipocyte lipolysis.

**Conclusion:**

We suggest that in T2D and CVD, increased production of CCL18 recruits and activates CD4^+^ T cells to secrete IFN-γ and TGF-β. This, in turn, promotes adipocyte lipolysis – a possible risk factor for cardiometabolic diseases.

**Supplementary Information:**

The online version contains supplementary material available at 10.1186/s12933-023-01803-w.

## Introduction

Individuals with obesity (BMI > 30 kg/m^2^) are at higher risk for cardiometabolic diseases, which includes cardiovascular disease (CVD) and/or type 2 diabetes (T2D) [1–3]). However, non-obese individuals (BMI < 30 kg/m^2^) can also be susceptible to multiple health complications like atherosclerosis, insulin resistance, T2D and coronary artery disease [4–7]. Systemic inflammation has been proposed as a risk factor for CVD and T2D also in non-obese individuals [8–10]. However, it is not known if the nature of inflammation differs between obese or non-obese individuals who are at risk for CVD or T2D.

The low-grade chronic inflammation is characterized by altered levels of circulating pro-inflammatory cytokines and immune cells [11–13]. In CVD, pro-inflammatory cytokines are upregulated systemically as well as locally in atherosclerotic plaques [14–17], where the balance between pro- and anti-inflammatory factors is affected [18]. Similarly, in obesity associated T2D, inflammatory cytokines and immune cells are dysregulated both in circulation as well as locally in white adipose tissue (WAT) [19–22]. In addition, several studies have shown that even non-obese individuals with CVD or T2D can have increased circulating pro-inflammatory cytokines [23–25],

Mounting evidence highlights the role of WAT in the development of cardiometabolic diseases [26, 27]. Changes in lipolysis and lipogenesis are observed in patients with insulin resistance and T2D [28] and WAT lipolysis is linked to variation in circulating lipids and cardiometabolic risk factors. Alterations in WAT fat cell metabolism become evident already in the healthy overweight state and does not deteriorate further in obesity or metabolic syndrome [29]. Some of these features, such as fat cell size are important factors for WAT inflammation [30]. The enlarged fat cells associate with increased WAT TNF-α secretion in healthy lean subjects as well as nonobese patients with T2D [31, 32]. On the other hand, WAT of non-obese men with T2D and enlarged fat cells was shown to display normal secretion of several inflammatory proteins [31].

Although several studies have suggested that even non-obese individuals with CVD/T2D can have increased circulating pro-inflammatory cytokines [23–25], the contribution of WAT inflammatory status to CVD and T2D in the non-obese individuals is unclear and this is the subject of the present investigation. We characterized the inflammatory status of abdominal subcutaneous WAT (scWAT) in non-obese individuals with or without CVD and/or T2D. Based on these characterizations, we aimed to define the possible factors behind the associations of WAT inflammation and adipocyte metabolism linking those to cardiometabolic diseases.

## Materials & methods

### Patient cohorts

Because of the complex nature of this study, six different patient cohorts were included. Most of the analysis in this study was performed in cohort 1, while data of other five cohorts have been reported previously [31, 33–36]. A summary of characteristics for each cohort is listed in Table 1. For cohort 1, subjects included in the Stockholm area were divided based on whether or not they had a diagnosis of diabetes and whether the coronary CT scan showed signs of coronary artery disease. In addition, a sub-study that involved adipose tissue biopsies and anthropometrical measurement by DEXA scan was carried out. This sub-study was approved by the Swedish ethical review authority and all subjects gave oral and written consent to be part of it. Cohort 1 consisted of 65 non-obese (BMI < 30 kg/m^2^) men and women classified into healthy, CVD, T2D and CVD with T2D groups as shown in Table 2 and additional file: Supplementary table S1. They were recruited from an ongoing population-based study, Swedish Cardio-Pulmonary bioImage Study (SCAPIS [htps://www.scapis.org]). Subjects with high fasting blood glucose (> 7 mmol/L) and HbA1c (≥48 mmol/mol) were classified as T2D. Status of coronary vascular bed was analyzed by computer tomography (CT) angiography and patients with coronary atherosclerosis at 7 or more sites were categorized as CVD. Patients with coronary atherosclerosis, high blood glucose and HbA1c were classified for T2D + CVD group. Except two women and one man with T2D and CVD + T2D, all patients in these two groups were treated with metformin. Other drugs used for the treatment of these patients are listed in Additional file: Supplementary Table S2. Patients in healthy and CVD groups underwent oral glucose tolerance tests and those with pathological outcomes were excluded. Fat cell volume (pL) was measured as described previously [31], where the light microscopy and a scale on the objective was used to measure the diameter of 100 cells. The average fat cell volume was calculated using the formula (π/6) X (3σ^2^xd + d^3^) (where d is the mean diameter and σ is the standard deviation of the diameter). Cohort 1 was used for immune cell profiling by flow cytometry, measurements of cytokine secretion, glycerol release from adipocytes and fat-pad, and correlation analyses of CCL18 with CD4^+^ T cell numbers. Cohort 2 was used to analyze the expression of CCL18 receptor, IFN-γ and TGF-β in FACS-sorted WAT cell populations [33]. Cohort 3 contained 40 obese insulin-sensitive and 40 obese insulin-resistant women [34]. Cohort 4 included 30 obese and 26 non-obese women with the broad range of metabolic parameters [35]. Cohort 5 contained 49 women where adipose tissue and metabolic data were available before the gastric bypass surgery [36, 37]. The latter three cohorts (3–5) were used to analyze association between CD4 and CCL18 expression. Cohort 6 consisted from 14 non-obese men with T2D, and 13 healthy men [31] and was used to measure the expression of the classical cytokines expressed CD4^+^ T cell subtypes.

**Table 1:** Summary of cohorts

Cohort	BMI (mean)	Summary of patient characteristics	Measurements available	Assay used in this paper	Reference as in the text
1	26.2	Non-obese men and women with T2D and/or CVD	WAT secretion samples, WAT phenotype, clinical metabolic parameters, serum measures, SVF	Measurement of cytokine secretions, immune cell phenotyping, correlation CCL18 with CD4 T cells, lipolysis. Correlations and comparisons between the four metabolic groups	Table 1
2	26.1	Obese and non-obese women	Global mRNA profiling in FACS sorted WAT cell populations and paired adipocyte samples	Expression of CCL18 receptor, IFN-γ and TGF-β in different adipose tissue resident cells	(33)
3	42.7	Obese IS and IR	Global WAT mRNA profiling, WAT phenotype, clinical metabolic parameters, serum measures	Correlation of CCL18 and CD4	(34)
4	33.1	Non-obese and obese IS and IR	Global WAT mRNA profiling, WAT phenotype, clinical metabolic parameters	Correlation of CCL18 and CD4	(35)
5	43	Obese (Before gastric bypass)	Global WAT mRNA profiling, WAT phenotype, clinical metabolic parameters	Correlation of CCL18 and CD4	(36)
6	26.2	Non obese men with and without T2D	WAT mRNA, WAT phenotype, clinical metabolic parameters	Expression of CD4 T cell cytokines	(31)

### Cytokine measurements in adipose tissue explants

The scWAT explant incubations were performed as previously described [31]. Adipose tissue explant incubation media was analyzed by Luminex multiplex immunoassay, employing customized 13-plex panel (ProcartaPlex ThermoFisher Vienna, Austria), which included adiponectin, IFN-γ, IL-1β, IL-1RA, IL-6, IL-8, MCP-1, MIF, ICAM-1, VCAM-1, IL-10, TNF-α and PAI-1. For Luminex multiplex immunoassay, 50 µl of each sample was analyzed according to manufacturer´s instructions. Data were collected using the Mapgix (Luminex xMAP™ Corporation, Austin, TX USA) and expressed as picograms per milliliter (pg/ml). Curve fit, alignment between the observed and expected concentrations in standard curves, as well as amount of counted beads (> 50) were used to validate the assays for individual cytokines.

In addition, AIF-1, CCL18, Angiopoietin-2 and C reactive protein (CRP) [38, 39] were analyzed by ELISA according to manufacturer instructions (Catalog numbers: DCL180D, DANG20 and DCRP00 respectively, Cloud Clone Corp SEC288Hu, R&D system Minneapolis, MN). Sensitivity and ranges of both platforms for adipose tissue cytokine measurements are indicated in Additional File 1: Supplementary Table S3.

### Cytokine quantification in serum

A venous blood sample for each subject was obtained for routine clinical measurements and serum was stored frozen for further analysis. Total of 47 samples were used for the cytokine measurements with n = 12 in healthy, n = 12 in CVD, n = 13 in T2D and n = 12 in CVD + T2D. Seven cytokines (IFN-γ, IL1β, IL1RA, IL-6, IL-8, IL-10, TNF-α) were measured using multiplex U-plex Mesoscale (MSD) platform (Mesoscale Discovery, Research Boulevard, Rockville, US), quantification of MIF was performed in 1-single U-plex due to the assay’s incompatibility with the above-listed cytokines. In both cases, 25 µl of each sample was analyzed according to the standard protocol, data collected with SECTOR Imager 6000 and analyzed with MSD Discovery Workbench^®^ software.

Cytokines sICAM-1, VCAM-1, MCP-1, PAI-1 and TNFα were measured with Luminex multiplex immunoassay using 5-plex panel (MagPix), because Mesoscale platform was not available for them. IL-10 was used as internal control to compare Luminex and Mesoscale platforms. Results obtained using both platforms correlated well (Additional File 1: Supplementary Fig. S1). AIF, CCL18, Angiopoietin-2, CRP and Adiponectin were measured using ELISA as described in 2.2. Sensitivity and ranges of Luminex, mesoscale and ELISA for cytokine measurements in circulation are indicated in Additional File 1: Supplementary Table S4.

### Cell cultures

Human adipose-derived stem cells (hASCs) were differentiated into adipocytes as previously described [40]. Jurkat T cells (clone E6-1, ATCC® TIB-152) were cultured in RPMI-1640 (Sigma-Aldrich, USA) medium with 10% FBS (Sigma-Aldrich), HEPES (Thermofisher Scientific) and penicillin and streptomycin (Thermofisher Scientific). To produce conditional media (CM), 5 × 10^5^ Jurkat T cells/500µl were treated with and without CCL18 (50ng/ml) (394-PA-050, R&D systems) for 24 h. Following this treatment, the CM was collected, centrifuged and the supernatant was added to the *in vitro* differentiated adipocytes for 24 h. For the cytokine-blocking experiments, the CM was collected as described above, treated with 10ng/ml IFN-γ (AF-246-SP, R&D systems) and 10ng/ml TGF-β (MAB2851-SP, R&D systems) blocking antibodies for 30 min at 37^o^C and then incubated with the *in vitro* differentiated human adipocytes for 24 h. Later, the media was collected, and cells were lysed for glycerol and protein measurements respectively.

### Measurements of cytokines in CM

Jurkat T cell CM was produced and collected as described above. IFN-γ and TGF-β were measured in CM using ELISA (Thermofisher Scientific EHFING and BMS249-4 respectively) according to manufacturer’s instructions.

### Flow cytometry

Cryopreserved stroma vascular fraction (SVF) was thawed, immediately washed with PBS/0.5% bovine serum albumin/2 mM EDTA buffer, filtered, and stained with antibodies (BD Biosciences, San Diego, CA; Bio-Techne, Abingdon, United Kingdom), as previously described [31]. Viability was assessed with 7-aminoactinomycin D (BD Biosciences). Total of 39 samples were used for immune phenotyping analysis using flow cytometry with n = 12 in healthy, n = 12 in CVD, n = 13 in T2D and n = 12 in CVD + T2D. 13-color flow cytometry antibody panel was applied to identify M1, M2 macrophages, CD4^+^, CD8^+^ T cells, B cells, regulatory T cells (Tregs), and adipocyte progenitors [31]. Combinations of cell surface markers used to identify each cell population are listed in Additional File 1: Supplementary Table S5. Antibody clones and their respective fluorophores are shown in Additional File 1: Supplementary Table S6. All FACS measurements were performed on BD FACS Aria™ Fusion cell sorter equipped with 405-nm, 488-nm, 561-nm, and 633-nm lasers and with Diva software (BD Biosciences). Data analyses were performed with FlowJo software (Tree Star, Ashland, OR). The purities of the sorted cell populations were typically above 95%. Fluorescence minus one control were used for all the antibodies to set marker-positive cell populations.

### Glycerol release

Lipolysis measurements in WAT adipocytes obtained by abdominal biopsies were performed according to a standard protocol [31, 41]. In brief, isolated fat cells / fatpad were incubated for 2 h at 37 °C in Krebs–Ringer phosphate buffer supplemented with glucose (8.6 µmol/ml), ascorbic acid (0.1 mg/ml) and BSA (20 mg/ml). Adipocytes were incubated in the absence (basal) or presence of increasing concentrations of noradrenaline or isoprenaline (stimulated). Basal and stimulated (noradrenaline and isoprenaline) lipolysis was measured as glycerol release and normalized per gram of fat tissue.

In addition, *in vitro* differentiated adipocytes were incubated with T-cell-derived CM, as described above. After 24 h media was collected from adipocytes and glycerol release was measured. Glycerol was quantified by the commercially available free glycerol reagent kit (F6428-40ml, Sigma-Aldrich) and Amplex ultra-red reagent (A36006, Invitrogen) as described previously [42]. Glycerol concentration was normalized to the protein amount in corresponding well measured by commercially available BCA kit (23,225, Thermofisher Scientific) [42].

### Gene expression analysis

RNeasy Micro Kit (Qiagen, Hilden, Germany) or NucleoSpin RNA kit (Macherey-Nagel, Duren, Germany) were used for RNA extraction according to manufacturer recommendations. RNA concentration was measured by NanoDrop 2000 spectrophotometer (ThermoFisher Scientific). Subsequently, 50 ng of total RNA was reverse transcribed using the iScript cDNA synthesis kit (Bio-Rad, Hercules, CA) according to manufacturer instructions. RT-qPCR was performed by using 1 ng of cDNA, SYBR green primers in 10µL reaction on the CFX96 Touch™ qPCR Detection System (Bio-Rad). List of primers used for RT-qPCR analyses are given in Additional File 1: Supplementary Table S*7*. Gene expression analysis in flow-cytometry sorted WAT cell populations was extracted form previously published microarray RNA profiling [33].

### Statistical analysis

A statistical power calculation was made on 17 WAT cytokines in four groups using g*power 3.1. According to the calculations, 44 individuals were needed to detect 50% difference at alpha 0.05 and power 96%. Multiple regression analyses were performed using JMP16 software (SAS Institute Inc., Cary, NC, USA) and the graphical illustrations were produced using GraphPad prism 7 (SCR_002798, La Jolla, CA). The normal distribution of the variables was verified using the Shapiro-Wilks test and logarithm transformation (log10) was employed where needed for variables to achieve normal distribution. The mean ± SD with minimal and maximal values were indicated for normally distributed values, whereas for non–normally distributed values, median–interquartile range with minimal and maximal values were indicated in the tables. One-way ANOVA with multiple comparisons was used to determine the significant changes between the groups of cytokines and immune populations. Unpaired parametric (t-test) was used to test the significance between two groups. Bubble plots showing the mean fold changes and 1-log 10 (p-values) of 17 cytokines and immune cells in the three metabolic disease groups in comparison to healthy group were generated using R. 1-log 10 (0.05) > 2.3 was considered statistically significant. UMAPs of WAT immune cells and CD4^+^ T cell populations were also generated using R [43]. Spearman correlation was used to evaluate association between two factors for non-normal distribution and Pearson correlation was used for all normally distributed factors. The volcano plot was generated from the multiple t Mann Whitney test representing the log transformed p values for each cytokine with respect to the rank difference between healthy and disease group. For multiple regression analysis using JMP 16, Fit model was used with CCL18 and BMI as dependent factors and CD4 as independent variable. P-value and β-coefficients for CCL18 and BMI regression with CD4 were shown. Data were presented as bar plots in the graphs with mean and SD. A probability level of 0.05 was considered statistically significant.

## Results

### Patient characteristics of cohort 1

Age and BMI matched individuals without obesity in cohort 1 were classified into healthy, CVD, T2D and CVD + T2D based on blood glucose, HbA1c levels and the presence of coronary atherosclerosis as shown in Table 2. As expected, patients with T2D and CVD + T2D had higher plasma glucose and HbA1c compared to healthy and CVD individuals (Table 2 and Additional File 1: Supplementary Table S1). There were no significant differences in fat cell volume, estimated subcutaneous adipose tissue (ESAT), % total fat and circulating triglycerides between any of the groups. Cholesterol concentrations were significantly lower in individuals with T2D and CVD + T2D compared to CVD only and the control groups, while log HOMA-IR was significantly higher in the groups with T2D compared to control and CVD-only individuals (Table 2). The lower cholesterol levels in subjects with diabetes may have been due to the use of statins (Additional file: Supplementary table S2) in these groups.

**Table 2:** Clinical parameters of cohort 1 For normally distributed values, mean ± SD with minimal and maximal values are shown. For non-normally distributed values (indicated with ^#^), median- interquartile range with minimal and maximal values are indicated. One-Way ANOVA was performed for each factor to determine the significant changes between the four groups. Significant values are indicated in bold. For HOMA-IR, statistical tests were performed after transforming the values in log.

Measure	Control (A)	CVD (B)	T2D (C)	CVD + T2D (D)	p-value ANOVA (ABCD)
**Gender** **F/ M**	8/9	9/9	4/9	6/9	
**Age** **years**	58 ± 3 (53–63)	61 ± 4 (55–68)	60 ± 4 (53–66)	60 ± 4 (52–65)	0.227
**BMI** **kg/m** ^**2**^	26 ± 2 (22–29)	26 ± 2 (22–29)	26 ± 2 (23–30)	27 ± 2 (24–30)	0.114
***% FAT DEXA***	34.3 ± 7.1 (21.2–45.6)	34.3 ± 9.8 (20.1–45.9)	31.2 ± 7.9 (14.2–41.4)	33.8 ± 6.1 (24–48)	0.793
***Abdominal scFAT (ESAT) grams***	1066–1607^#^(970–2446)	1272 ± 480 (689–2168)	900 ± 440 (313–1819)	1043 ± 610 (277–2763)	0.086
**P-glucose mmol/l**	5.6 ± 0.4 (4.9–6.7)	5.5 ± 0.3 (5.1–6.4)	7.5 ± 1.6 (5.6–10.6)	6-8.8 ^#^ (4.9–20.2)	**< 0.0004**
**HbA1c mmol/mol**	35 ± 5 (24–44)	37 ± 2 (33–41)	46 ± 9 (34–67)	42–65 ^#^ (40–84)	**< 0.0001**
**Fat cell volume pl**	547 ± 90 (438–770)	406–610 ^#^ (7–1295)	538 ± 178 (336–900)	609 ± 107 (422–758)	0.410
**TG** **mmol/l**	1.1–1.7 ^#^ (0.8–3.4)	0.8–1.2 ^#^ (0.4–3)	0.8–1.6 ^#^ (0.7–3.7)	0.8–2.7 ^#^ (0.5–6.8)	0.232
**Cholesterol mmol/l**	5 ± 0.7 (4–7.2)	5.1 ± 1.1 (3.2–7.3)	4.1 ± 1.0 (2.6–6.5)	4.1 ± 1.1 (2.6–6.7)	**0.006**
**HOMA-IR**	1.3 ± 0.46 (0.6–2.4))	1.2 ± 0.5 (0.5–2.5)	2.5 ± 1.9 (0.4–7.3)	1.2 ± 4.7 (0.3–17.6)	**0.025**

### WAT CCL18 is significantly upregulated in cardiometabolic disease groups

**Fig. 1:**
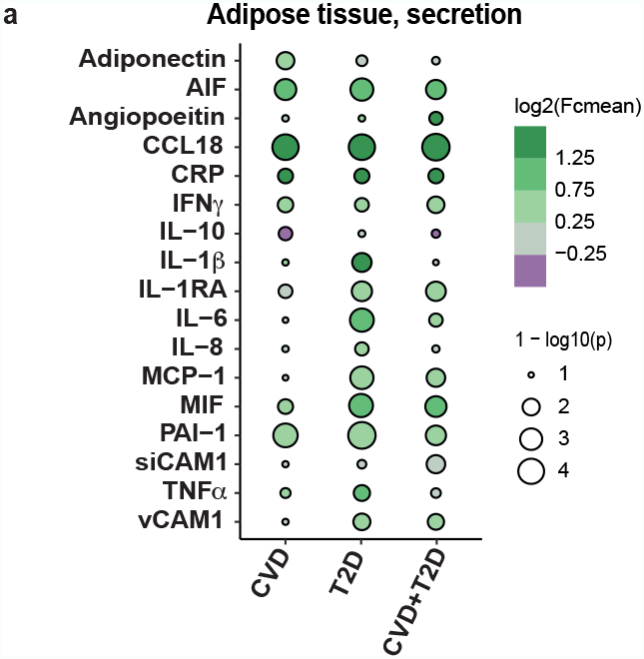
Adipokine secretion in WAT of the metabolic groups of non-obese individuals Bubble plots representing the fold changes (FC) and significance of 17 WAT-secreted adipokines in the three metabolic diseases groups CVD (n = 12), T2D (n = 13) and CVD + T2D (n = 12) compared to healthy controls (n = 12). Size of the circles corresponds to the strength of the p-values that were calculated by one-way ANOVA with multiple comparisons using the raw secretion values (pg/ml). 1-log10(p-value) > 2.3 are considered statistically significant. The color of the circles corresponds to the mean fold change of the secretion levels for each disease group compared to healthy controls. Cohort 1 was used.

In order to map the release of adipokines from WAT and in circulation, we analyzed 17 different pro- and anti-inflammatory secreted proteins in adipose tissue explants and in serum samples. Seven of them showed differential secretion levels between the groups in WAT. IL-1RA, IL-6 and MCP-1 were significantly higher in patients with T2D compared to healthy controls (Fig. 1 and Additional File 1: Supplementary Fig. S2). MIF was upregulated in individuals with T2D and CVD + T2D, whereas AIF was upregulated in CVD-only and T2D-only individuals (Fig. 1 and Additional File 1: Supplementary Fig. S2). PAI-1 and CCL18 were upregulated in all three disease groups in comparison to healthy group (Fig. 1a and Additional File 1: Supplementary Fig. S2). Among these two, CCL18 showed highest fold change in all three disease groups compared to healthy controls (Fig. 1). These differences were not reflected in the circulating cytokines (Additional File 1: Supplementary Fig. S3).

In summary, among 17 profiled adipokines, CCL18 showed the highest significant upregulation in WAT obtained from the individuals with cardiometabolic diseases, therefore it was selected for further analysis.

### WAT CD4^+^ T cells are upregulated in metabolic disease groups

**Fig. 2:**
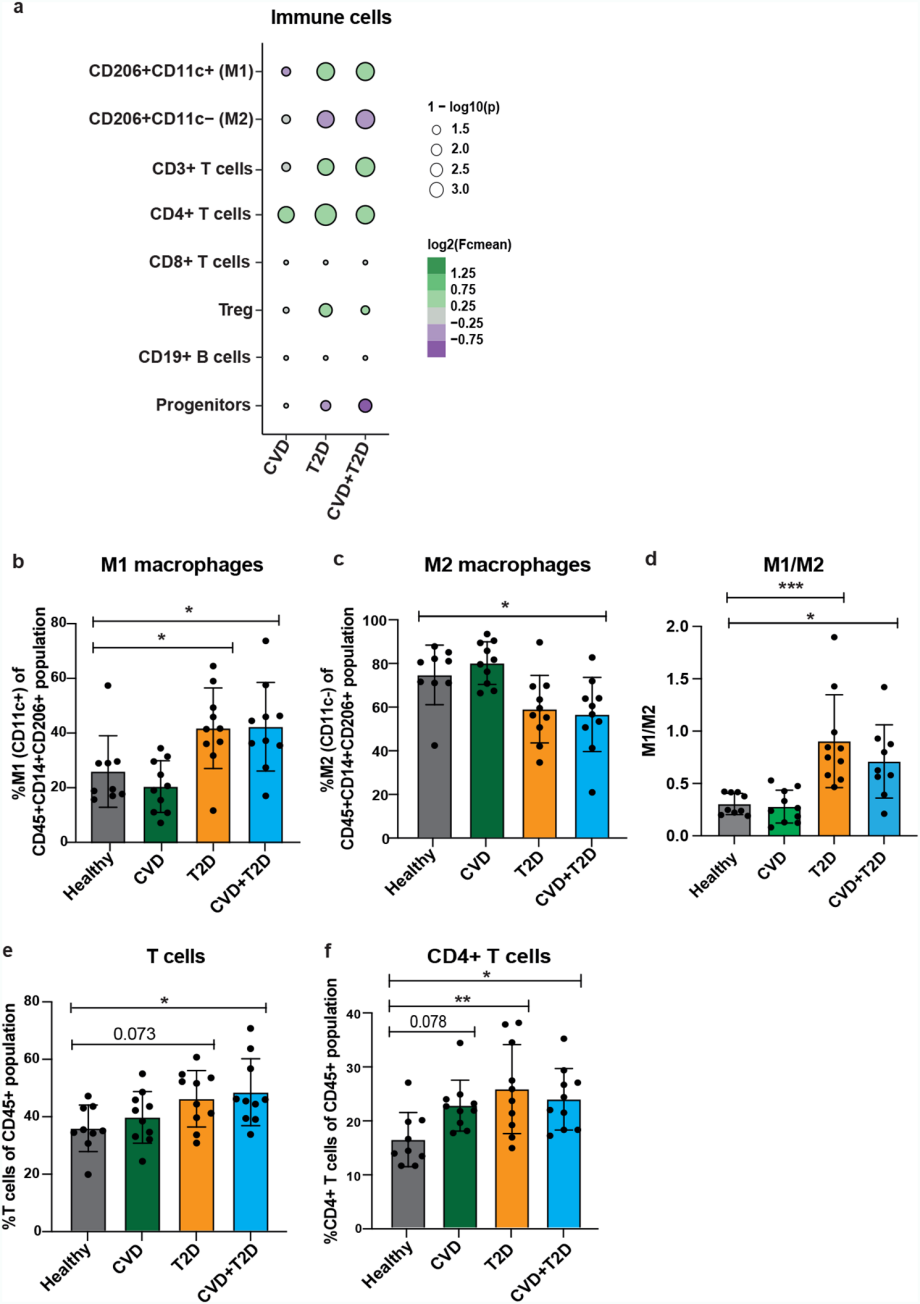
Immune cell profiling in WAT SVF of cohort 1 (a) Bubble plots representing the fold changes in the frequencies of WAT SVF immune cell populations for the three metabolic diseases groups CVD (n = 10), T2D (n = 10) and CVD + T2D (n = 10) compared to healthy controls (n = 9). Size of the circles corresponds to the strength of the p-values that were calculated by one-way ANOVA with multiple comparisons. 1-log10(p-value) > 2.3 are considered significant. The color of the circles corresponds to the mean fold change in the frequencies of each immune cell population in the disease group compared to healthy controls. The percentage of M1 (CD206^+^CD11c^+^) (b) and M2 (CD206^+^CD11c^−^) (c) macrophages in total WAT macrophage population (CD14^+^CD206^+^), M1/M2 ratio (d), the percentage of total CD3^+^ T (e) cells and CD4^+^ T cells (f) (in total immune cell population (CD45^+^). Grey, green, yellow, and blue bars represent healthy, CVD, T2D and CVD + T2D respectively. One-way ANOVA with multiple comparisons was used in b to f. * p < 0.05 and ** p < 0.01. Cohort 1 was used in (a) - (f).

Previous publications [39] and additional analysis of published single-cell sequencing data [43], suggested that CCL18 was highly expressed in myeloid cell populations (Additional File 1: Supplementary Fig. S4a). Among those it was particularly enriched in three clusters annotated as M2 macrophages and Lipid Associated Macrophages (LAMs) [43] (Additional File 1: Supplementary Fig. S4b). In addition, mRNA profiling in FACS-sorted WAT cell populations confirms that CCL18 is most enriched in adipose tissue macrophages (Additional File: Supplementary Fig. S4c). Therefore, we further applied a multi-color flow cytometry analysis on WAT SVF (Additional File 1: Supplementary Fig. S5a) to examine if changes in cytokine secretion are associated to the possible differences in the immune cell-population frequencies in adipose tissue. The obtained data indicated that in the groups including individuals with T2D there was a significant shift towards M1 phenotype in macrophages (Fig. 2a). Upregulation of the M1 macrophage (CD14^+^CD206^+^CD11^+^) population was observed in individuals with T2D (Fig. 2a, b). M2 macrophage frequency was lower (Fig. 2c), and therefore M1/M2 ratio was increased (Fig. 2d) in the T2D groups compared to healthy controls. Thus, we could speculate that there is a shift of WAT-resident macrophages from anti- to pro-inflammatory state in the non-obese individuals with T2D. Interestingly, WAT immune cell populations were least affected in CVD group compared to the other metabolic groups.

In addition, total CD3^+^ T cells were significantly upregulated in individuals with CVD + T2D (Fig. 2a, e) and showed borderline significance (p = 0.073) in the group with T2D compared to healthy controls. A subpopulation of T cells - CD4^+^ T cells - was significantly upregulated in T2D, CVD + T2D groups while a borderline significance (p = 0.078) was detected for CVD group compared to healthy controls (Fig. 2a, f). There were no significant differences in CD8^+^ T cells, B cells, Tregs and adipocyte progenitor cells in any of the groups (Fig. 2a and Additional File 1: Supplementary Fig. S5b).

Taken together, we found changes in both macrophage populations and T cell frequencies in cardiometabolic disease groups, where CD4^+^ T cell were increased in all disease groups.

### CCL18 correlates positively with CD4^+^ T cells in WAT

**Fig. 3:**
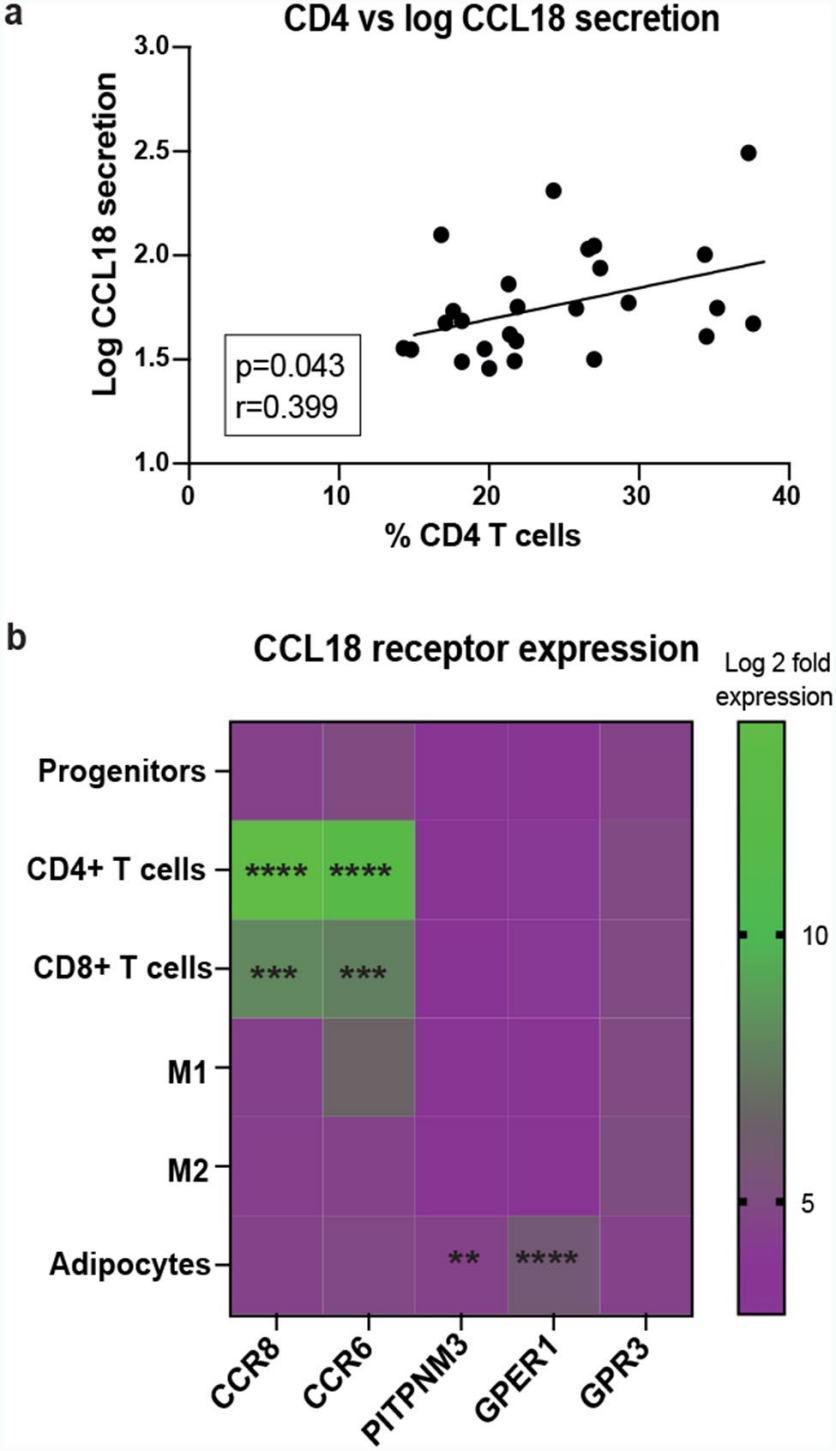
CD4 T cells as possible responders to CCL18. a Pearson correlation (r- and p-values) of CCL18 secretion levels from WAT explants and the percentage of WAT CD4^+^ T cells in cohort 1 (n = 28). b Relative mRNA expression levels (log2) of CCL18 receptors CCR8, CCR6, PITPNM3, GPER1 and GPR3 in different WAT populations (RNA expression profiling by microarray in Cohort 2). Two-way ANOVA with multiple comparisons was performed in (b). **p < 0.01, *** p < 0.001 and **** p < 0.0001.

Data described above demonstrated that CCL18 and CD4^+^ T cells were upregulated in the three cardiometabolic disease groups compared to healthy controls. It is well-known that CCL18 is a chemoattractant for T cells [44], therefore we next investigated whether WAT CCL18 levels are associated with the frequency of adipose CD4^+^ T cells. WAT CCL18 secretion correlated significantly with the percent of CD4^+^T cell in scWAT (Fig. 3a). In addition, multiple regression analysis of CCL18 and CD4 mRNA in three patient cohorts showed that the expression of these two genes correlated significantly and independently of BMI (Table 3).

**Table 3:** Multiple regression analysis of CCL18 and CD4 in three different cohort Multiple regressions in cohorts 3, 4 and 5 using CCL18 and BMI as independent variables. p-values and standardized β coefficients for CCL18 and BMI are shown in relation to the dependent variable CD4. Significant correlations are indicated in bold.

CD4	CCL18		BMI	
	β -coeff	p-value	β -coeff	p-value
Cohort 3	**0.7197**	**< 0.0001**	0.1919	0.0555
Cohort 4	**0.8832**	**< 0.0001**	-0.0326	0.7280
Cohort 5	**0.3115**	**0.0081**	**0.3115**	**< 0.0001**

To further investigate possible crosstalk between WAT CCL18 and CD4^+^T cells, we analyzed the expression of CCL18 receptors (*CCR8, CCR6, PITPNM3, GPER1* and *GPR3*) in WAT cell populations. Although *PITPNM3* and *GPER1* were found on adipocytes, *CCR6* and *CCR8* were significantly higher expressed on CD4^+^ T cells (Fig. 3b) compared to all other WAT populations, indicating that CCL18 might act through *CCR6* and *CCR8* on CD4^+^ T cells.

In summary, our data suggests that upregulation of CCL18 might attract CD4^+^ T cells to scWAT and activate them under cardiometabolic diseases. Therefore, we aimed to further investigate how this could affect adipose tissue metabolism and its possible association with CVD and T2D.

### CCL18 increases lipolysis through CD4^+^ T cells

**Fig. 4:**
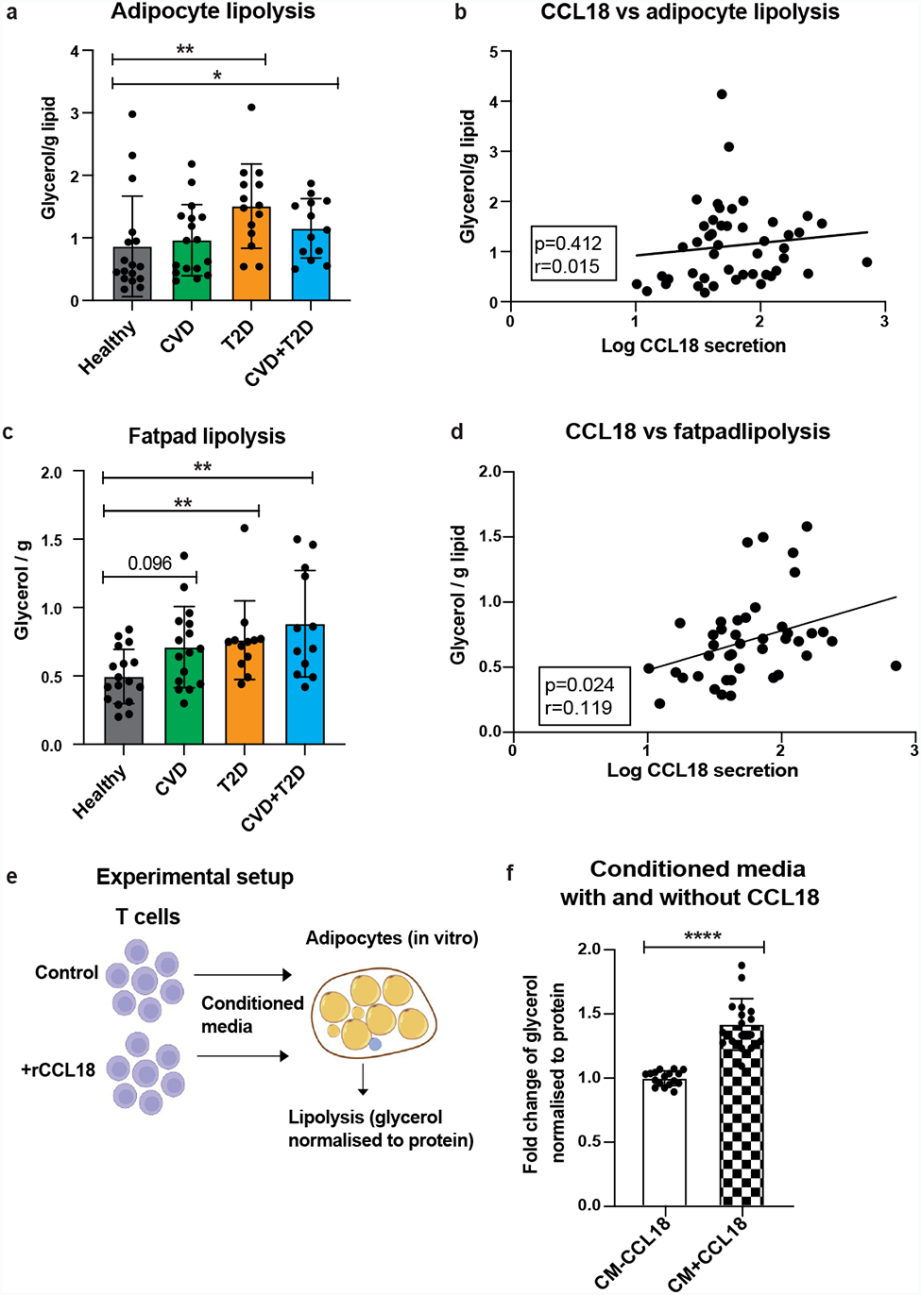
CCL18 induce adipocyte lipolysis via activation of T cells (a) Adipocyte lipolysis measured by glycerol µmol/g of lipid/2 hours in different metabolic groups (n = 16 for healthy, n = 16 CVD, n = 12 T2D and n = 12 CVD + T2D). (b) Pearson correlations (r- and p-values) of CCL18 secretion and adipocyte lipolysis (n = 47). Fat pad lipolysis (c) and correlation of CCL18 secretion with fat pad lipolysis (d) (n = 42). (e) Schematic representation of the experimental set-up to evaluate T cell role in CCL18-mediated lipolysis induction. (f) *in vitro* adipocyte lipolysis measured as a FC in the glycerol release normalized to protein amount in adipocytes treated with the CM from T cells (n = 17). Empty and checkered bars represent the CM from T cells treated without and with CCL18 treatment, respectively. One-way ANOVA with multiple comparisons was used in (a) and (c) and the unpaired t-test was used in (f). *p < 0.05, **p < 0.01 and ****p < 0.0001. Cohort 1 was used in a – d. *In vitro* differentiated hASCs were used in f.

Lipolysis of scWAT adipocytes has been shown to correlate with fasting plasma lipid levels contributing to the development of cardiometabolic diseases, such as CVD and T2D [28, 45–47]. *Ex vivo* adipocyte lipolysis was upregulated in T2D groups when compared to healthy controls (Fig. 4a) and CCL18 secretion did not correlate to the basal lipolysis rate of purified scWAT adipocytes (glycerol/g lipid of adipocytes) (Fig. 4b). However, glycerol release from intact fat pad was significantly increased in T2D and CVD + T2D groups, also showing a borderline significance in CVD group (p = 0.096) (Fig. 4c). In addition, fat pad lipolysis significantly correlated to WAT CCL18 secretion (Fig. 4d).

Furthermore, only the basal fat pad lipolysis was upregulated and correlated to CCL18 expression in cardiometabolic disease groups. There was neither a significant correlation of CCL18 secretion to stimulated lipolysis of purified adipocytes (like maximum response to Isoprenaline-isomax or Noradrenaline -NAmax) nor there were any changes in the stimulated lipolysis rate in three cardiometabolic disease groups compared to healthy controls (Additional File 1: Supplementary Fig. S6).

Similar to our previous study [39], we could not observe a direct CCL18 effect on lipolysis in mature adipocytes (Additional File 1: Supplementary Fig. S7). Therefore, we hypothesized that CCL18 might act via regulation of CD4^+^ T cell recruitment and phenotypic change, which subsequently could affect adipocyte lipolysis.

To test this hypothesis, we designed an experiment where the T cells were treated with and without CCL18 for 24 h and the resulting CM from the T cells was added on the *in vitro* differentiated human adipocytes (Fig. 4e). This showed that lipolysis (glycerol release normalized to protein) was significantly upregulated in adipocytes incubated with the CM obtained from T cells treated with CCL18 compared to control CM acquired without CCL18 (Fig. 4f). These data suggested that CCL18 can upregulate basal lipolysis via CD4^+^ T cells.

### IFN-γ and TGF-β are secreted by T cells stimulated by CCL18

**Fig. 5:**
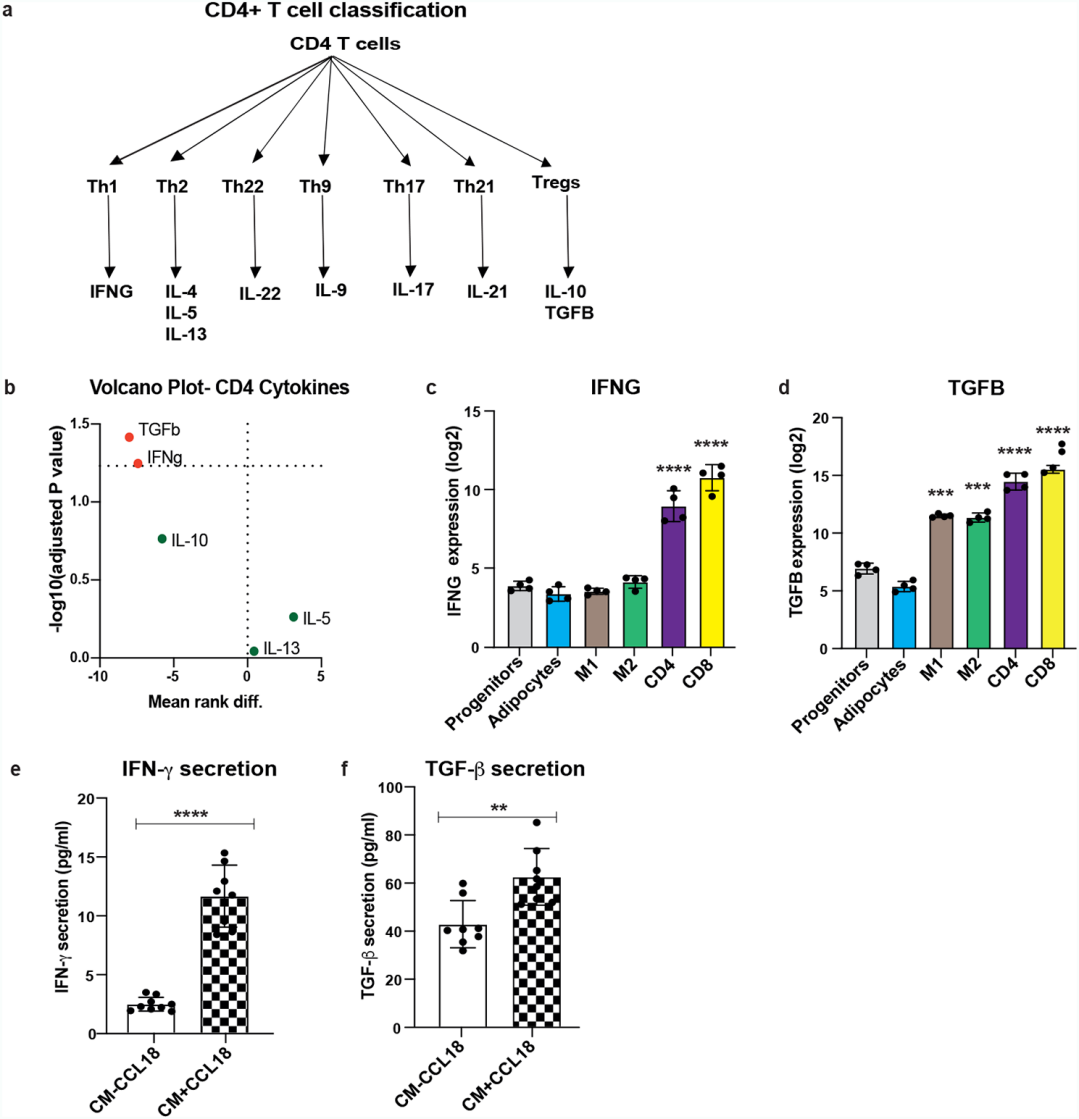
IFN-γ and TGF-β as possible factors mediating CCL18-induced CD4^+^ T cell response A diagram of cytokine secretions from different subsets of CD4^+^ T cells based on literature. b The volcano plot, representing the inverse log of the p-values (-log10) for each cytokine with respect to the rank difference between healthy (n = 13) and T2D (n = 13) groups (cohort 6). The volcano plot analysis was generated from the multiple Mann-Whitney test for five cytokines (TGFB, IFNG, IL-10, IL-13, and IL-5). Positively upregulated cytokines (IFNG and TGFB) during T2D are indicated in red. *TGFB* (c) and *IFNG* (d) expression in different WAT SVF populations (cohort 2). IFN-γ (e) and TGF-β (f) secretion in the CM from T cells treated without (n = 9) and with (n = 8) CCL18. One-way ANOVA with multiple comparisons was performed in (c) and (d). Unpaired t-test was performed in (e) and (f). **p < 0.01; *** p < 0.001 and **** p < 0.0001.

We further hypothesize that CCL18-activated T cells secrete factors, which in turn upregulate adipocyte lipolysis. The subtypes of CD4^+^ T cells are classified into six groups based on their production of cytokines (Fig. 5a) and the expression of the characteristic lineage-defining transcription factors [48].

In search for lipolysis-upregulating factors, we first measured expression of ten cytokines (*IL-4, IL-22, IL-9, IL-17, IL-21, TGFB, IFNG, IL-10, IL-13* and *IL*-*5*) secreted by CD4^+^ T cell subpopulations from cohort 6. Expression of five of the cytokines (*IL-4, IL-22, IL-9, IL-17*, and *IL-21*) were below the detection limits in scWAT. Figure 5b shows the volcano plot, representing the log transformed p values for each cytokine (*TGFB, IFNG, IL-10, IL-13*, and *IL*-5) with respect to the rank difference between healthy and T2D group indicated that *IFNG* and *TGFB* (in red) were the only two cytokines significantly upregulated in non-obese T2D individuals compared to control group (Fig. 5b). In addition, the expression of *IFNG* and *TGFB* were highly enriched in T cells (Fig. 5c, d) compared to other cell populations of scWAT.

Next, we analyzed if CCL18 treatment affects secretion of IFN-γ and TGF-β in T cells. Measurements of these cytokines in T cell CM demonstrated that both IFN-γ and TGF-β release were significantly upregulated in CM + CCL18 compared to CM-CCL18 (Fig. 5e, f).

Altogether, these results suggested that IFN-γ and TGF-β could be the factors secreted by T cells and responsible for upregulation of adipocyte lipolysis.

### Blocking IFN-γ and TGF-β inhibits lipolysis

**Fig. 6:**
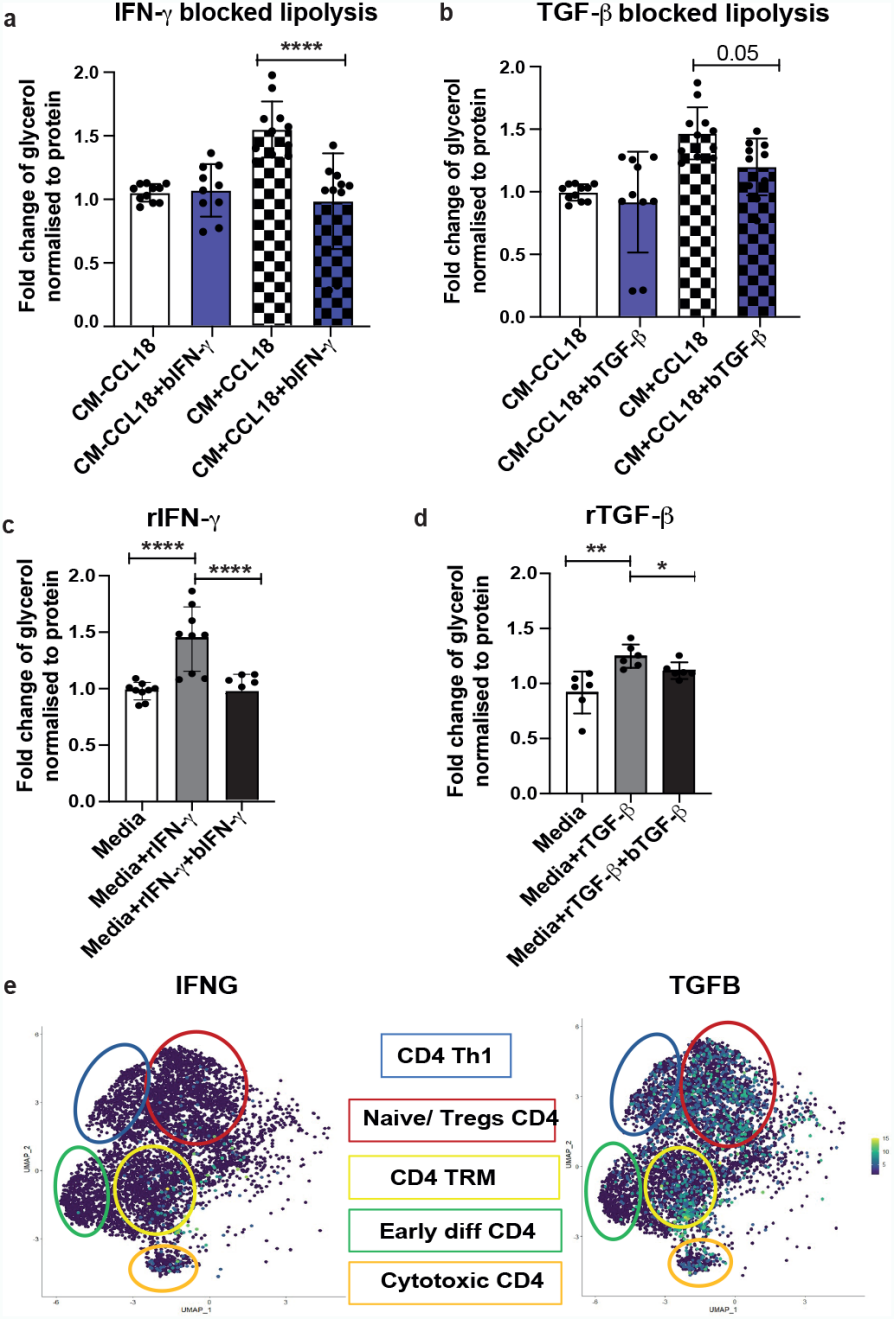
TGF-β and IFN-γ mediates CCL18/CD4 T cell-induced lipolysis FC of *in vitro* adipocyte lipolysis induced by CM obtained from T cells stimulated with and without CCL18, in the presence and absence of IFN-γ (a) or TGF-β (b) blocking antibodies (n = 10). FC of the lipolysis in adipocytes stimulated with and without rIFN-γ (c) in the presence or absence of blocking IFN-γ antibodies (n = 9); and treatment with rTGF-β combined with TGF-β blocking antibodies (n = 6) is shown in (d). Statistical significance was evaluated by one-way ANOVA with multiple comparisons. * p < 0.05, ** p < 0.01, *** p < 0.001 and **** p < 0.0001. UMAPs for *IFNG* and *TGFB* expression in CD4^+^ T cell clusters are shown in (e). Dots are colored according to the expression levels, going from darker purple for low expression to yellow for high expression. Circles represent different clusters. Circle color corresponds to the color of the populations represented in the boxes.

We further inhibited action of these two cytokines using blocking antibodies. Incubation of T cell CM with the IFN-γ blocking antibodies prior to the treatment of adipocytes, abolished lipolytic effect of CM + CCL18 (Fig. 6a). Inhibition of TGF-β, also attenuated lipolytic effect of this cytokine, showing a borderline significance (p = 0.0504) in CM + CCL18 with blocking compared to CM + CCL18 without blocking antibodies (Fig. 6b). No significant differences were observed between CM-CCL18 and CM + CCL18 + blocking antibodies, indicating that the blocking antibodies have attenuated the lipolysis to basal levels of control cells. In addition, lipolysis was upregulated by direct treatment of adipocytes by recombinant (r) IFN-γ and rTGF-β. And also, this effect was inhibited by IFN-γ and TGF-β blocking antibodies (Fig. 6c, d).

It was previously observed that IFN-γ and TGF-β are secreted by two distinct subsets of CD4^+^ T cells like T helper 1 cells and regulatory T cells respectively [49–51]. To further explore this notion and possibly find a T cell subset that might secrete both these cytokines, we employed previously published single cell sequencing data, visualizing them by high resolution UMAPs of CD4 T cell populations in WAT [43]. TGFB was expressed by almost all encircled subsets of CD4^+^ T cells (Fig. 6e, right), whereas IFNG was expressed only in two clusters (Fig. 6e, left), which were annotated as cytotoxic and tissue resident memory CD4^+^ T co-expressing genes for activation markers like HLA-DR and CD69 [43]. Even though the IFNG expression was weaker compared to TGFB, we found that both these genes were co-expressed in cytotoxic and TRM CD4 T cells populations, indicating that these particular populations might be associated with CCL18 function.

## Discussion

In this study, we demonstrated that the non-obese patients with different cardiometabolic diseases, like CVD and/or T2D, showed significant upregulation of WAT CCL18 among the 17 different tested cytokines. Although there were alterations in macrophage and T cell population proportions in some cardiometabolic disease groups, CD4^+^ T cells were the only immune population, which showed an upregulation in all three disease groups compared to healthy controls. Furthermore, we showed that CCL18 and CD4^+^ T cells were significantly correlating to each other independent of BMI and that CCL18 mediated CD4^+^ T cell-dependent lipolysis upregulation through IFN-γ and TGF-β.

Although patients with CVD were confirmed to have atherosclerosis at 7 or more sites by MRI, their plasma TG and cholesterol levels were similar to healthy controls. Even though TGs are an important risk factor for CVD development, the correlations between TG levels and CVD are controversial. In contrast to the studies showing significant associations between CVD and TGs [52, 53], there are reports invalidating the positive association between TGs and CVD risk when an adjustment was made for other lipoproteins [54, 55]. In addition to TGs, weak positive or inverse correlations have also been shown between cholesterol and atherosclerosis [55, 56]. These studies therefore claimed that it is impossible to consider the high blood cholesterol or TGs as the major risk factors of CVD since people with low levels of both both (cholesterol and TGs) become just as atherosclerotic as individuals with high levels and their risk of suffering from CVD is the same or higher. Our data also does not support high levels of TGs and cholesterol being CVD-predicting risk factors.

Furthermore, we did not observe differences in fat cell volume in subjects with cardiometabolic diseases compared to healthy individuals. In obesity, fat cell hypertrophy in subcutaneous and visceral adipose tissue during obesity are associated with their pernicious metabolic profile [57–59]. In addition, we showed previously that non-obese men with T2D had larger fat cells compared to healthy controls [31]. The difference between our previous and current study could be due to the inclusion of both men and women in the latter. Unfortunately, we lacked statistical power to investigate fat tissue morphology in women and men separately. To obtain enough power to detect meaningful differences between groups in cohort 1 we had to pool data from both sexes. Like other studies, we saw an upregulation of HbA1c and plasma glucose in patients with T2D [60–62].

CCL18 is a chemokine displaying the features of an inflammatory cytokine [63]. The production of this chemokine has been shown to be induced by infectious agents like HIV and induced by other cytokines like IL-4, IL-10, and IL-13 [64, 65]. CCL18 upregulation has been reported in a number of diseases, including HIV infection, atherosclerosis, pulmonary fibrosis, T2D, and in various cancers like breast, cervical, lung, and ovarian [15, 66–68]. This cytokine has also been used as a biomarker for lung diseases like systemic sclerosis and idiopathic pulmonary fibrosis [69–71]. Regarding cardiometabolic disease, CCL18 has been shown to be expressed in atherosclerotic plaques [16, 72]. Few publications have shown the association of CCL18 with T2D in adipose tissue, but the mechanism for these associations remained unknown [39, 73]. In contrast to Versteylen et al. [68] who showed a positive correlation of plasma CCL18 with disease progression of coronary artery disease (CAD), we did not find an upregulation of circulating CCL18 in individuals with CVD, while we found a WAT-specific upregulation of CCL18 in CVD and T2D. This difference could be due to the more pronounced severity of the CAD in their cohort where 50% of the patients had an obstructive CAD diagnosis, which was likely to result in more severe inflammatory phenotype.

CCL18, being a chemoattractant, can recruit CD8^+^ and CD4^+^ T cells and especially Tregs [74, 75]. Su et al. reported the adherence of CD4^+^ T cells to tumor slices in proportion to CCL18 presence and CCL18-dependent infiltration of the adoptively transferred human naïve CD4^+^ T cells in tumors of humanized mouse [76]. Our current study demonstrates that total CD4^+^ T cell population is upregulated in the disease groups compared to controls, which follows pattern of increased CCL18. However, we did not see any changes in WAT Treg or CD8^+^ population in cardiometabolic disease, indicating that these cell populations might not be recruited by CCL18 in human WAT. In general, CD4^+^ T cells are known factors regulating WAT function. Th1 and Th17 have been shown to be upregulated during obesity and promoting inflammation and insulin resistance in adipose tissue, whereas Th2 and Tregs have been shown to play a role in immune and metabolic homeostasis [77]. Most of the subsets of CD4^+^ T cells (except Th1, Th17) have been associated with anti-inflammatory WAT phenotype [78]. However, our current data indicate that in non-obese individuals CD4 + cells correlate positively with HOMA-IR, TGs, glucose and insulin (data not shown). Furthermore, we suggest that CCL18 might mediate a phenotypic change of CD4^+^ T cells inducing production of IFN-γ and TGF-β. Although earlier studies indicate that these two factors are produced by two distinct T cell subpopulations, Th1 and Tregs respectively [49–51], meta-analysis of single cell transcriptomics data suggested that there might be a specific subpopulation of T cells expressing both these factors. It is also possible that CCL18 is activating several distinct T cell populations, which we could not identify with the FACS panels used, and which can be addressed by the future studies.

Impaired lipolysis has been shown to associate with both T2D and CVD [45, 47, 79]. Increased lipolysis could lead to the release of excess free fatty acid and that in turn could be a major risk factor for both T2D and CVD [79]. IFN-γ has been reported to promote adipocyte lipolysis via stimulation of prostaglandin synthesis [80]. To the best of our knowledge, the role of TGF-β in lipolysis is less studied. We observed that IFN-γ and TGF-β promote lipolysis, which was confirmed by blocking the cytokines using blocking antibodies. Apart from the role of these cytokines in lipolysis, increased WAT TGF-β can also impair adipogenesis, increase fibrosis, induce lipid catabolism and production of inflammatory factors [81]. Similarly, IFN-γ has been shown to attenuate insulin signaling, lipid storage and differentiation in human adipocytes [82].

In this study, we were able to profile 17 cytokines in WAT and serum, as well as to phenotype major WAT SVF cell populations with a 13-color flow cytometry panel using small amounts (1–2 g) of clinical samples, which is a clear strength of the study. However, detection of some factors (including IFN-γ) by Luminex had higher detection threshold compared to ELISA. Therefore, it is important to note that there still might be additional differences in the secretion of WAT cytokines in cardiometabolic conditions, which could not be detected by Luminex. In addition, we couldn’t examine visceral tissue from this non-obese patient cohort, which could provide additional important information, but was not feasible due to ethical reasons. Furthermore, it would have been an advantage to perform all measurements in the same larger cohort, but the nature of the study made it impossible. It was not possible either to validate our data and to investigate further causal effects using *in vivo* animal models as CCL18 has no homolog in rodents [83, 84].

In conclusion, we suggest that CCL18 is upregulated in WAT of non-obese subjects with cardiometabolic conditions, which in turn attracts and activates CD4^+^ T cells to secrete IFN-γ and TGF-β promoting adipocyte lipolysis and possibly contributing to disadvantageous metabolic phenotype.

### Electronic supplementary material

Below is the link to the electronic supplementary material.

Supplementary Material 1

## Data Availability

Data are available from the corresponding author for any interested researcher who meets the criteria for access to confidential data and upon reasonable request.
