# Correction: Adipose tissue specific CCL18 associates with cardiometabolic diseases in non-obese individuals implicating CD4^+^ T cells

**DOI:** 10.1186/s12933-023-01961-x

**Published:** 2023-08-30

**Authors:** Narmadha Subramanian, Kaisa Hofwimmer, Beatriz Tavira, Lucas Massier, Daniel P. Andersson, Peter Arner, Jurga Laurencikiene

**Affiliations:** Lipid Laboratory, Unit of Endocrinology, Dept. of Medicine Huddinge, Karolinska Institutet, 141 86 Stockholm, Sweden

**Correction: Cardiovascular Diabetology (2023) 22:84** 10.1186/s12933-023-01803-w

This commentary highlights a few points that have come to the attention of the authors after the study was published and discussed with prof. Mikael Rydén, the head of the unit for Endocrinology, Department of Medicine Huddinge at Karolinska Institutet, where the study [1] was performed. Mikael Rydén is also a holder of the ethical permit for the clinical cohort 1 described in the study. We think that the information below can be important for the readers of Cardiovascular Diabetology and for future publications on this cohort.

- The cohort described in the paper (cohort 1) is a part of Swedish population-based SCAPIS cohort containing more than 30 000 individuals. In this cohort, coronary arteries were assessed as described in Bergström et al. (Circulation 2021;144:916–929). In the present study, patients with coronary atherosclerosis in 2 (not 7 as mistakenly stated) or more segments were categorized as CVD. This will not change the results or the message of the study.
- In our study (cohort 1), we have divided patients into four categories; two of them including individuals with type 2 diabetes and two of them diabetes-free. We do not mention or discuss glucose intolerance in our study and regard Diabetes as a real disease and not glucose intolerance. However, we would like to clarify that two of the patients in the control group and two in the CVD group had values of oral glucose tolerance tests (OGTT) slightly above the control levels (highest being 8.4 mmol/L) (normal OGTT is below 7,8 mmol/L, Diabetes value is above 11 mmol/L) and one individual in a control group had impaired fasting glucose level of 6.7 mmol/l (according WHO, impaired fasting glucose levels are 6.1–6.9 mmol/l, diabetes levels are > 7 mmol/l). When performing the study, we considered a “pathological value” of fasting glucose and oral glucose load as a diabetes value and anything below as a non-diabetic value, which is also stated in the text of the paper. Removing these patients from the analysis did not affect the conclusions reached by the study. However, after the concern was raised by prof. M. Rydén, we would like to note this information in the commentary.
- The statistical analysis is based on data obtained from a total of 63 individuals (as stated in the paper). These were included among the first participants enrolled in the study, that were matched for age and BMI and had samples required for chosen analysis. Part of the major methodologies used in the paper (adipokine profiling and flow cytometry) are not feasible in larger cohorts, which was also appreciated by the reviewers. Recruitment of cohort 1 continued after our adipokine profiling was performed and includes almost 200 individuals collected by prof. M. Rydén. We are aware that measures available for the entire cohort might differ from those reported by us due to a stronger statistical power.
- Lastly, we would like to acknowledge prof. Mikael Rydén for providing valuable information and comments on the published study. In addition, we would like to acknowledge prof. Claes Frostell for unbiased evaluation of the ethical aspects of the study and a scientific advice.

Bergström et al. Circulation 2021;144:916–929.
