# Supplementary material for: Adipose tissue specific CCL18 associates with cardiometabolic diseases in non-obese individuals implicating CD4+ T cells
Source: Cardiovasc Diabetol. 2023 Apr 12;22:84. doi: 10.1186/s12933-023-01803-w (PMC10099890; doi:10.1186/s12933-023-01803-w)
Supplement: Supplementary file 1 — Supplementary Material 1 [file 12933_2023_1803_MOESM1_ESM.pdf]

<u>Figure S1</u> Comparison of Luminex and Mesoscale platforms

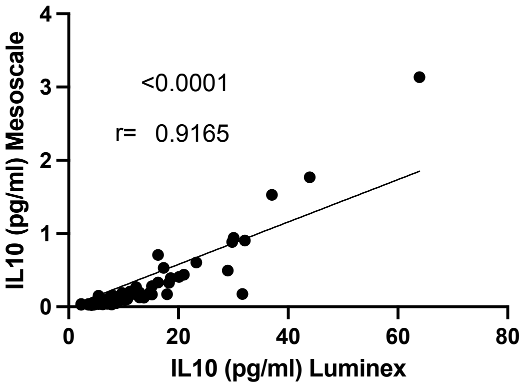

Figure S1. Pearson correlation of IL-10 WAT secretion (pg/ml) measured by Luminex and mesoscale in cohort 1 (n=75).

<u>Figure S2</u> WAT adipokine secretions

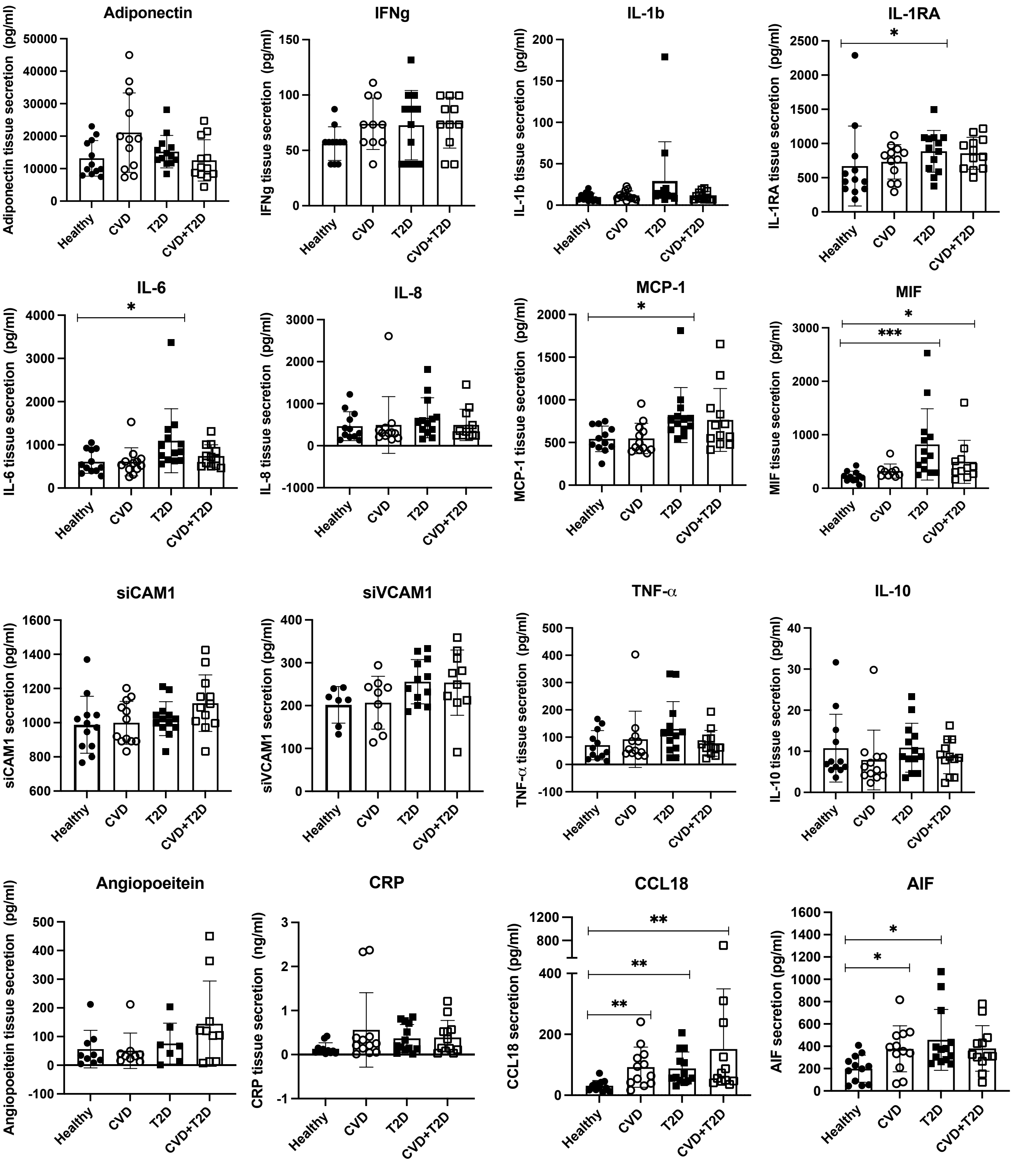

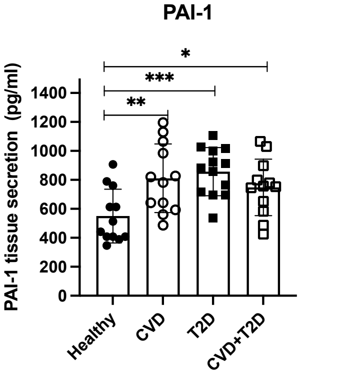

Figure S2. Quantification of seventeen adipokines secreted from WAT explants in four groups of individuals (Cohort 1); healthy (n=12), CVD (n=12), T2D (n=13) and CVD+T2D (n=12) . All values are pg/ml. One-way ANOVA with multiple comparisons was used to define significant differences between healthy and the disease groups. * p<0.05, ** p<0.01, *** p<0.001.

Figure S3 Cytokine secretions in serum

a

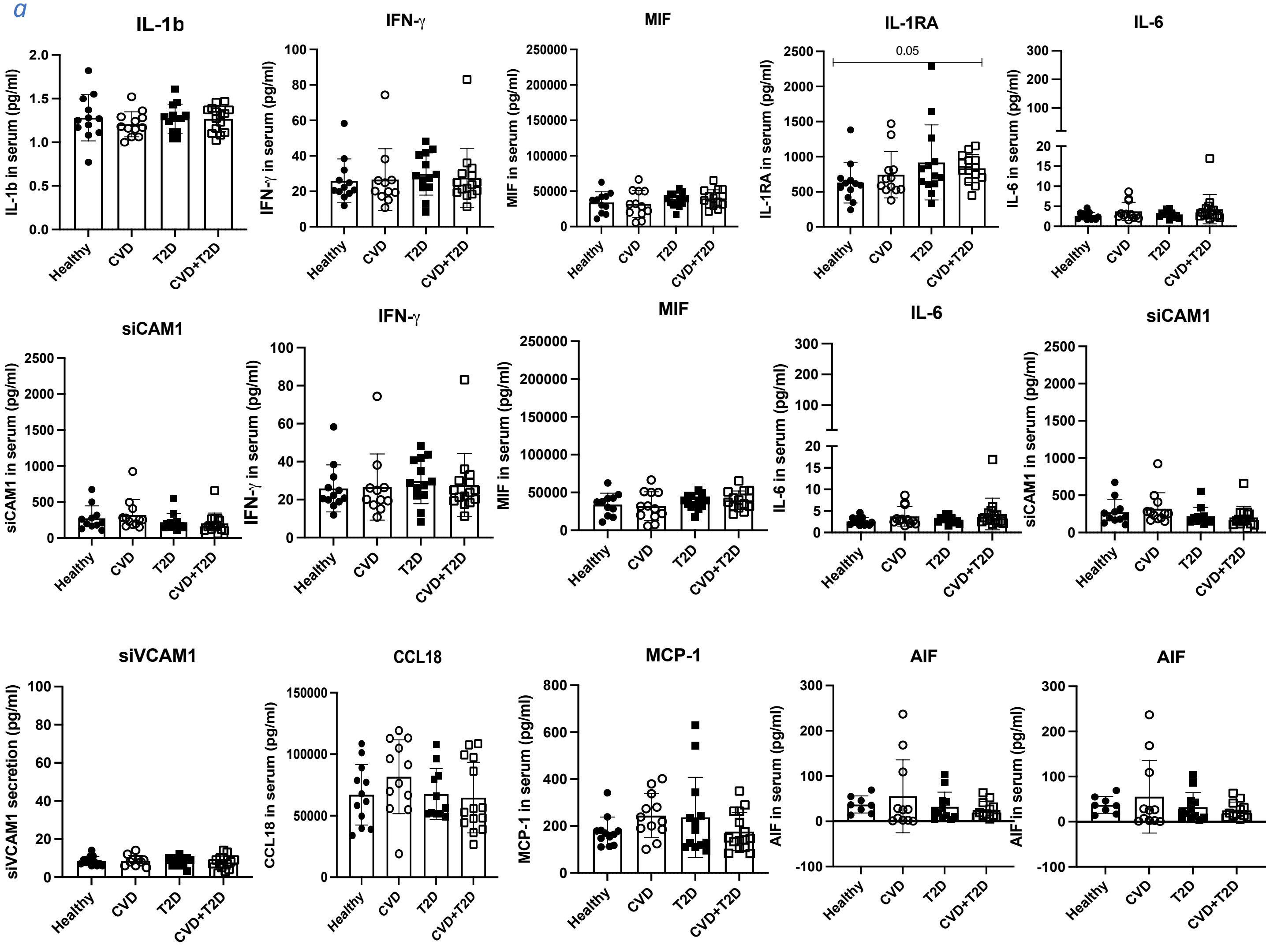

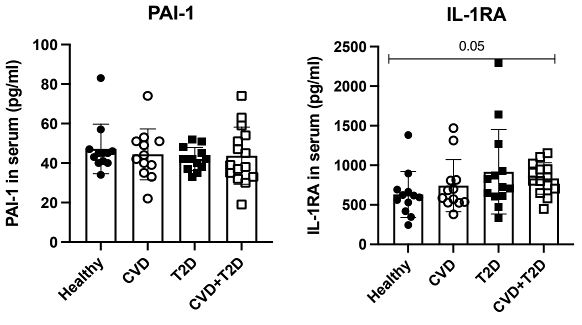

b

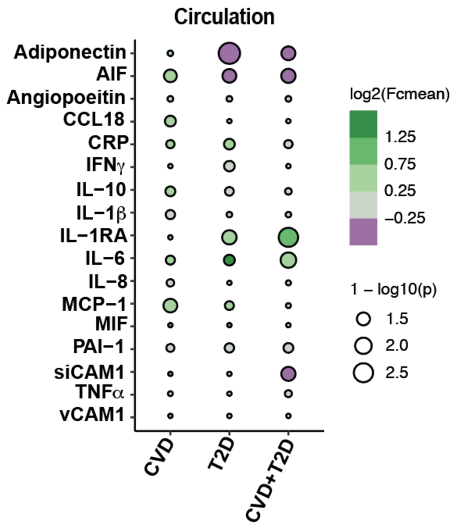

Figure S3. a. Quantification of seventeen adipokines in serum of four groups of individuals (Cohort 1); healthy (n=12), CVD (n=12), T2D (n=13) and CVD+T2D (n=15) . All values are pg/ml. One-way ANOVA with multiple comparisons was used to define significant differences between healthy and the disease groups. * p<0.05. b. Bubble plots representing the fold changes (FC) and significance of 17 circulating adipokines shown in (a). Size of the circles corresponds to the strength of the p-values that were calculated by one-way ANOVA with multiple comparisons using the raw secretion values (pg/ml). 1-log10(p-value) > 2.3 are considered statistically significant. The color of the circles corresponds to the mean fold change of the secretion levels for each disease group compared to healthy controls.

Figure S4 CCL18 expression in WAT immune cells

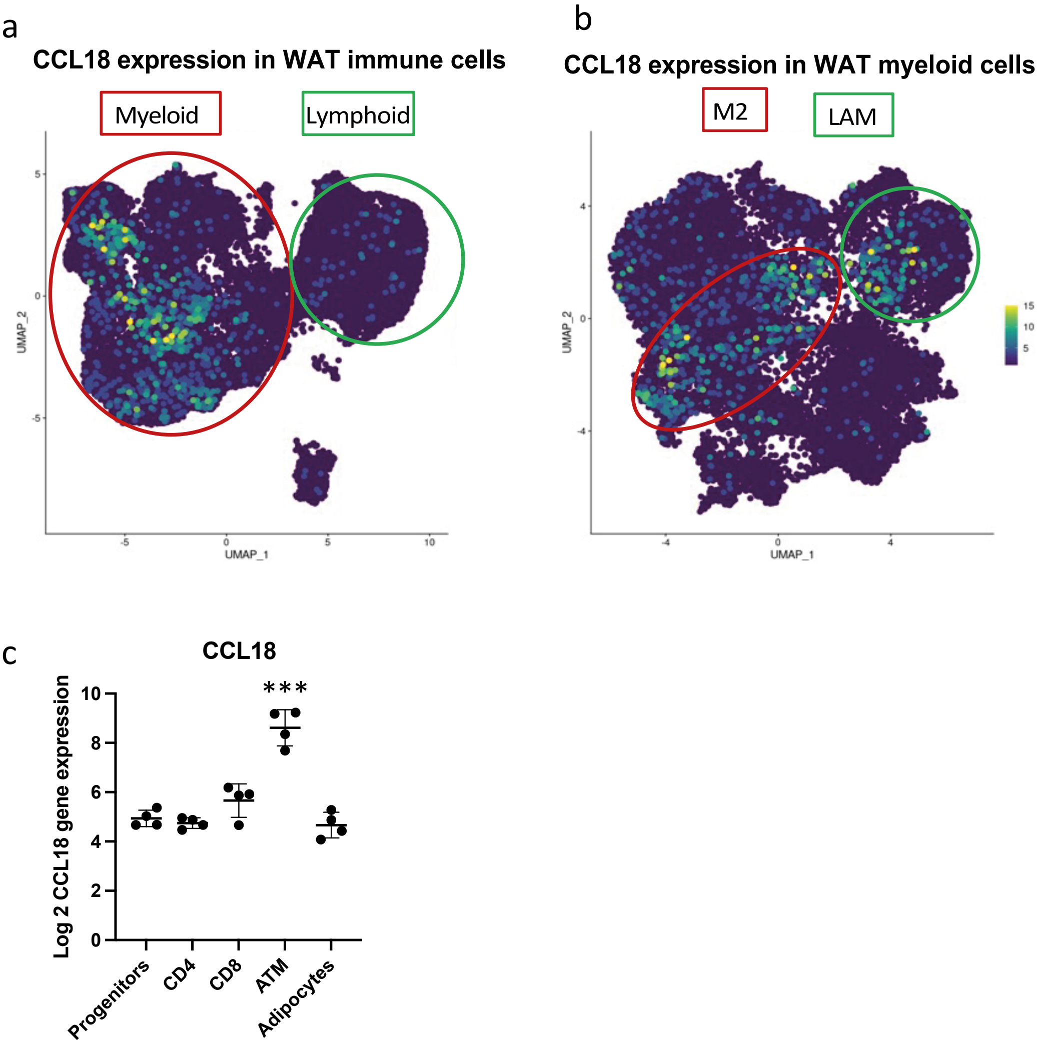

Figure S4. UMAPs generated by the additional analysis of the already published single cell sequencing data (Massier et al., nat commun. 2023) show the CCL18 expression in WAT immune cells (a) and in the WAT myeloid cells (b). Red and green circles correspond to myeloid and lymphoid populations in the left and M2 and LAM populations in the right respectively. (c) CCL18 expression in the different FACS sorted immune populations. Cohort 2 was used for this analysis. One-way ANOVA with multiple comparisons was used to define significant differences between the different immune populations.

Figure S5 Flow cytometry gating strategy and cell population frequences

a

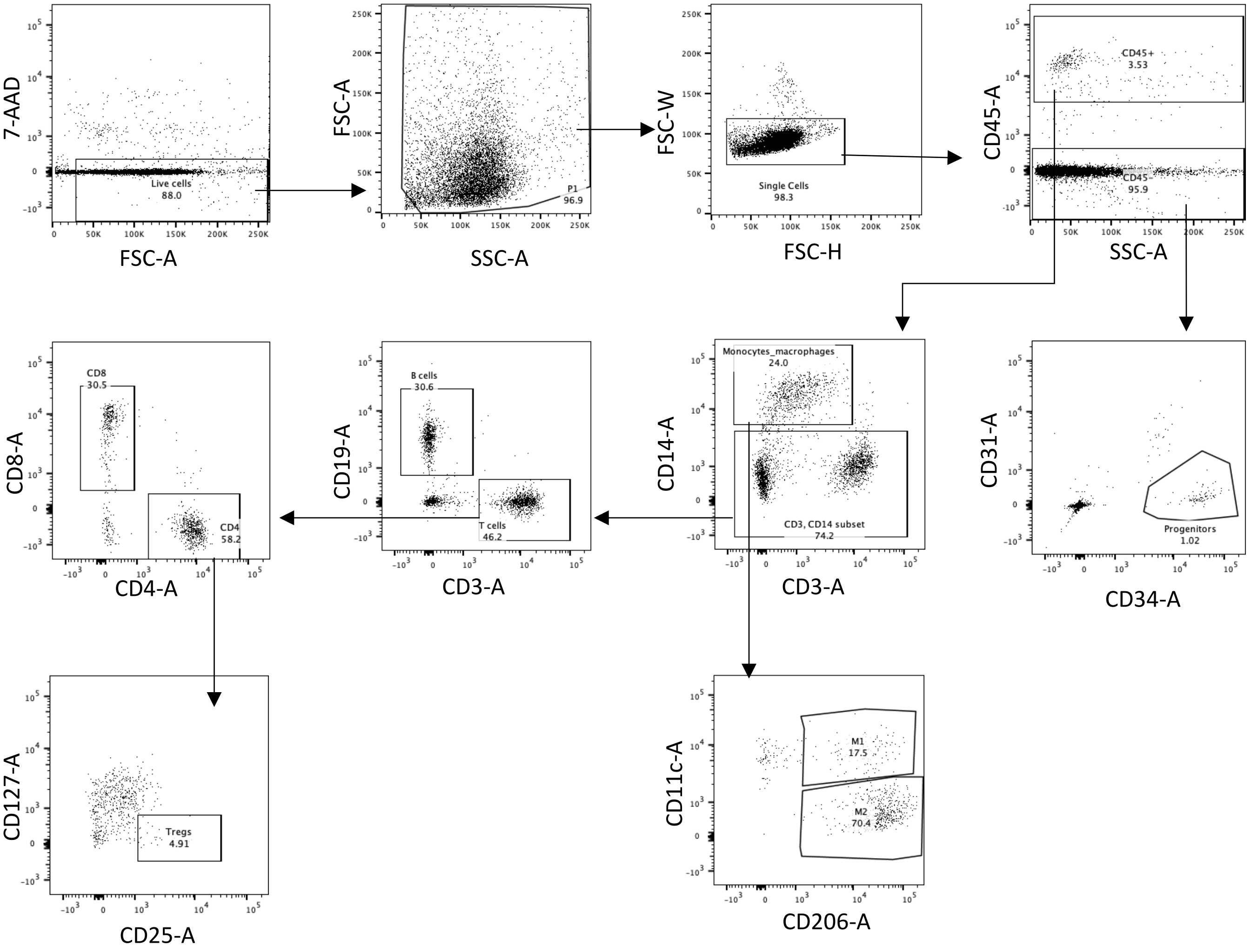

b

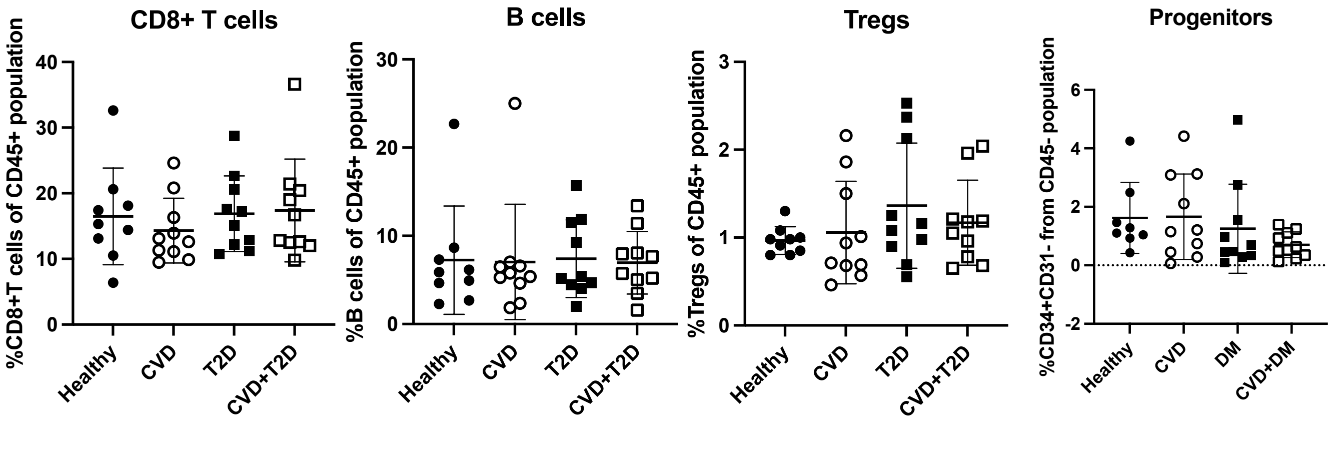

Figure S5. a. The flow cytometry gating strategy of WAT SVF cell populations using 13-color panel. **b.** Frequency of $CD8^+$ T, B, T_{regs} and adipocyte progenitor cells for all the four groups (Cohort 1); CVD (n=10), T2D (n=10) and CVD+T2D (n=10) and healthy controls (n=9). One-way ANOVA with multiple comparison test was used to evaluate the significance between healthy and the disease groups. Black and white circles and black and white squares are used to represent healthy, CVD, T2D and CVD+T2D respectively.

Figure S6 Stimulated lipolysis

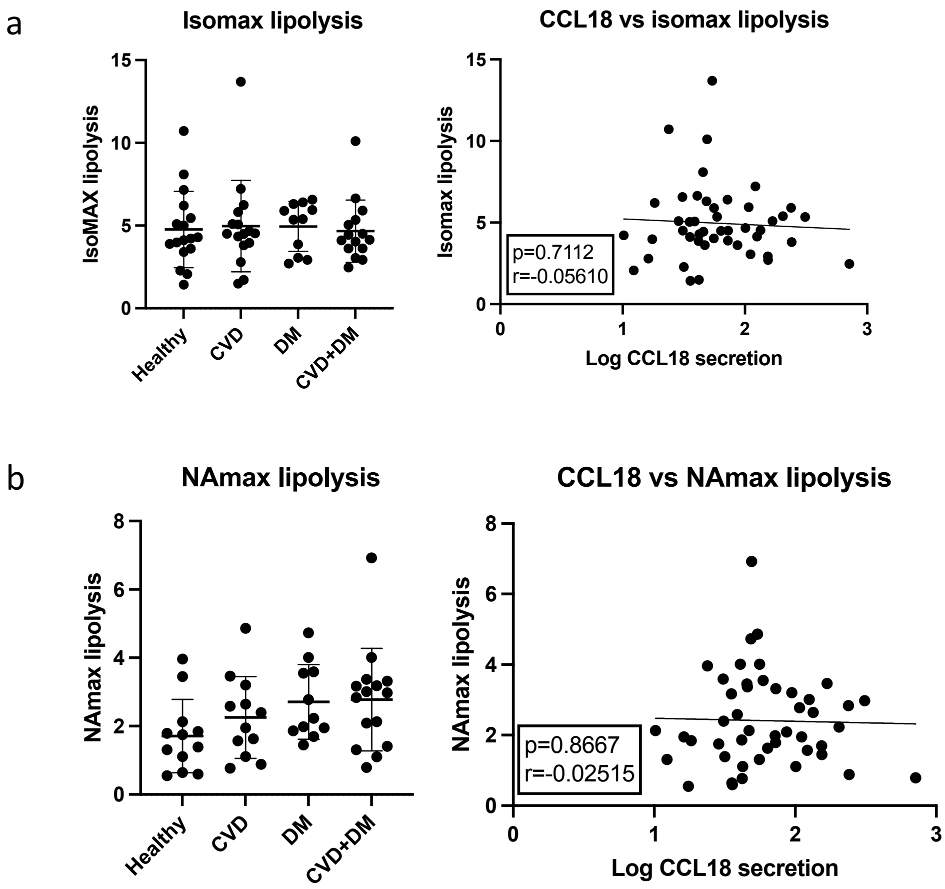

Figure S6. Maximum response to isoprenaline (Isomax) (a) and Noradrenaline (Namax) (b) was measured by performing titrations of isoprenalin and noradrenaline and glycerol measurements in purified *ex vivo* adipocytes in four metabolic groups of cohort 1; healthy (n=17), CVD (n=16), T2D (n=11) and CVD+T2D (n=15). One-way ANOVA multiple comparison test was used to test the significance between healthy and disease groups. Pearson correlations (r- and p- values) of isomax and NAmax lipolysis response to CCL18 WAT secretion (n=47) are shown in the right panels of (a) and (b) respectively.

Figure S7 Lipolysis in *in vitro* differenciated adipocytes stimulated by rCCL18

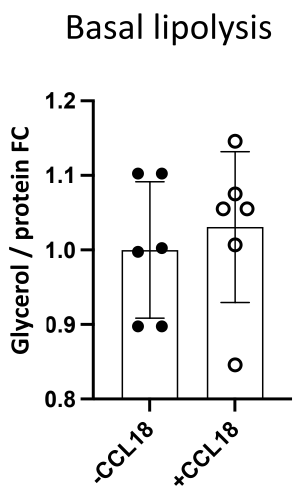

Figure S7. Glycerol release from *in vitro* differentiated mature adipocytes treated with or without rCCL18 (t-test, n=6).

Table S1 Comparisons of clinical parameters in the four metabolic groups

Measure	p-value (one-way ANOVA multiple comparison)					
	CVD vs Contr	T2D vs Contr	CVD+T2D vs Contr	T2D vs CVD	CVD+T2D vs CVD	CVD+T2D vs T2D
**Age, years**	0.180	0.585	0.507	0.9246	0.939	0.999
**BMI, kg/m^2**	0.954	0.949	0.344	0.999	0.135	0.167
**% body fat DEXA**	>0.999	>0.999	0.167	>0.999	>0.999	>0.999
**ESAT (gram)**	>0.999	0.184	0.489	0.407	>0.999	>0.999
**P-glucose, mmol/l**	0.830	**0.040**	**0.003**	**0.030**	**0.002**	0.688
**HbA1c, mmol/mol**	0.879	**0.003**	**<0.0001**	**0.022**	**<0.0001**	0.086
**Fat cell volume, pl**	0.900	0.999	0.756	0.960	0.335	0.740
**TG, mmol/l**	0.702	0.997	0.731	0.854	0.167	0.678
**Cholesterol, mmol/l**	0.972	0.097	0.087	**0.036**	**0.029**	0.999
**Log HOMA-IR**	0.732	0.340	**0.042**	0.176	**0.027**	0.732

Contr - Healthy control group; Significant values are indicated in bold.

Table S2: Medications given to the individuals in cohort 1

	Healthy	CVD	T2D	CVD+T2D
Betablockers	0	1	1	3
ACE-inhibitors/ARB	0	4	6	5
Thiazide diuretics	0	1	0	0
Calcium channel blockers	0	0	1	5
Statins	1	3	5	9
Ezetimibe	0	1	0	0
Metformin	0	0	7	12
DPP4-inhibitors	0	0	1	1
SGLT2-inhibitors	0	0	0	1
Sulphonylureas	0	0	2	1
Insulin	0	0	1	2

Number of subjects taking different medications are given in each column. ACE = Angiotensin Converting Enzyme, ARB Angiotensin Receptor Blocker, DPP4 Dipeptidyl peptidase, SGLT2 Sodium GLucose coTransporter-2

Table S3 Detection range of Luminex and ELISA assays used to measure secreted proteins in WAT explants

ProcartaPlex Mix & Match Human 13-plex (Mapgix)			
**Number**	**Target name**	**Bead region**	**Standard concentration range (pg/ml)**
1	Adiponectin	57	194600 – 11.88
2	IFNγ	43	48200 – 2.94
3	IL-1β	18	9000 – 0.55
4	IL-1RA	38	124200 – 7.58
5	IL-10	28	10100 – 0.62
6	IL-6	25	38300 – 2.34
7	IL-8	27	10500 – 0.64
8	MCP-1	51	17200 – 1.05
9	MIF	67	850 – 0.05
10	PAI-1	35	96100 – 5.87
11	sICAM-1	73	443200 – 27.05
12	sVCAM-1	74	32300 – 1.97
13	TNFα	45	34200 – 2.09
**ELISA**			**Standard concentration range (pg/ml) (ng/ml) ***
14	AIF-1	-	2000 – 31.2
15	CCL-18	-	1200 – 18.8
16	Angiopoitein-2	-	3000 – 46.9
17	CRP*	-	50 – 0.8*

Table S4 Detection range of Mesoscale, Luminex and ELISA assays used for cytokine measurements in circulation (serum)

U-plex U-plex Mesoscale (MSD) 7-plex			
**Number**	**Target name**	**Bead region**	**Standard concentration Range (pg/ml)**
1	IFNγ	-	27400 – 1.67
2	IL-1β	-	3630 – 0.22
3	IL-1RA	-	4630 – 0.13
4	IL-10	-	3610 – 0.22
5	IL-6	-	2060 – 0.13
6	IL-8	-	2010 – 0.12
7	TNFα	-	3650 – 0.22
	**U-plex Single-plex (SECTOR Imager 6000)**		
8	MIF	-	135500
	**ProcartaPlex Mix&Match Human 5-plex (Mapgix)**		**Standard concentration Range (pg/ml)**
9	sICAM-1	73	582000 – 35.52
10	sVCAM-1	74	38000 – 2.32
11	MCP-1	51	14600 – 0.89
12	PAI-1	35	104000 – 6.35
12	TNFα	45	40800 – 2.49
	**ELISA**		**Standard 1 concentration (pg/ml) (ng/ml) ***
13	AIF-1	-	2000 – 31.2
14	CCL-18	-	1200 – 18.8
15	Angiopoitein-2	-	3000 – 46.9
16	CRP*	-	50 – 0.8*
17	Adiponectin*	-	250 – 3.9*

Table S5 Cell surface marker combinations defining different WAT SVF cell populations

Population	Gating
B cells	$CD45^{+}CD14^{-}CD3^{-}CD19^{+}$
T cells	$CD45^{+}CD14^{-}CD3^{+}CD19^{-}$
CD4+ T cells	$CD45^{+}CD14^{-}CD19^{-}CD3^{+}CD4^{+}$
CD8+ T cells	$CD45^{+}CD14^{-}CD19^{-}$ $CD3^{+}CD8^{+}$
M1 macrophages	$CD45^{+}CD3^{-}$ $CD14^{+}CD206^{+}CD11^{+}$
M2 macrophages	$CD45^{+}CD3^{-}$ $CD14^{+}CD206^{+}CD11^{-}$
Regulatory T cells	$CD45^{+}CD14\text{-}CD19^{-}$ $CD3^{+}CD4^{+}CD25^{+}CD127^{-}$
Adipocyte progenitors	$CD45^{-}CD31^{-}CD34^{+}$

<u>Table S6</u> Antibodies used for Flow Cytometry

Marker	Fluorophore	Clone	Source	Catalog no.	Dilution	RRID
CD45	AF700	HI30	BD	560566	1:80	AB_1645452
CD14	BV711	MΦP9	BD	563373	1:80	AB_2744290
CD11c	Pe-Cy7	B-ly6	BD	561356	1:80	AB_10611859
CD206	PE	19.2	BD	555952	1:5	AB_396248
CD34	PE-CF594	563	BD	562449	1:5	AB_11152083
CD31	FITC	WM59	BD	560904	1:5	AB_10562392
CD3	BV786	SK7	BD	563799	1:5	AB_2744384
CD4	BV605	RPA-T4	BD	562658	1:5	AB_2744420
CD8	APC-H7	SK1	BD	560273	1:5	AB_1645482
CD19	BV650	SJ25C1	BD	563227	1:5	AB_2744313
CD25	BV421	M-A251	BD	562443	1:5	AB_11153864
CD127	AF647	HIL-7R-M21	BD	560905	1:5	AB_10563899
7-AAD			BD	559925	1:1000	

RRID – Research resource identifiers

Table S7 Primers used for RT-qPCR analysis
(gene and primer sequences, SyBr (Sigma-Aldrich))

**Gene name**	**Forward primer**	**Reverse primer**
IL5	AACTGTGCACTGAAGAAATC	CTAGGAATTGGTTTACTCTCC
IL13	ATCACCCAGAACCAGAAG	ATGCAAGCTGGAAAACTG
IL17A	CATTGGTGTCACTGCTAC	TCGGTTGTAGTAATCTGAGG
IL17F	TGGAATTACACGTCACTTG	GAACGGAATTCATGGAGATG
IL21	CCAGAAGATGTAGAGACAAAC	ATAAGAATCACATGAAGGGC
IL22	CTGTGAGCTCTTTCCTTATG	AACGTCTGTGTTGTTATCAG
IFNG	GGTAACTGACTTGAATGTCC	TTTTCGCTTCCCTGTTTTAG
IL9	CATCAACAAGATGCAGGAAG	ATCAGTGGGTATCTTGTTTG
IL4	TCACATTGTCACTGCAAATC	CCTTCTCAGTTGTGTTCTTC
IL4 Seq 2	ACTTTGAACAGCCTCACAGAG	TTGGAGGCAGAAAGATGTC
IL4 Seq 3	CCACGGACACAAGTGCGATA	CCCTGCAGAAGGTTTCCTTCT
TGFB1	CCCACAACGAAATCTATGAC	TGTATTTCTGGTACAGCTCC
CCL18	CTATACCTCCTGGCAGATTC	CTCTCTTGGTTAGGAGGATG
CD4	TGTCCTCACCGGTTTC	ACAATGGCAAATTGTAGAGG
CD8	CTCTACCCTCCAACAAAAG	TTTTGTAGAGATGGGGGTTC
